# Double stigma in mental health: epilepsy and mental illness

**DOI:** 10.1192/bjo.2020.58

**Published:** 2020-07-13

**Authors:** Marco Mula, Kenneth R. Kaufman

**Affiliations:** Institute of Medical and Biomedical Education, St George's University of London, UK; and the Atkinson Morley Regional Neuroscience Centre, St George's University Hospitals NHS Foundation Trust, UK; Robert Wood Johnson Medical School, Rutgers University, New Brunswick, USA; and Institute of Psychiatry, Psychology, and Neuroscience, King's College London, UK

**Keywords:** Epilepsy, stigma, double stigma, depression, psychiatric disorders

## Abstract

**Background:**

Epilepsy and mental illness share similar problems in terms of stigma, as a result of centuries of superstition, ignorance and misbeliefs. Stigma leads not only to discrimination and civil and human rights violations but also to poor access to healthcare and non-adherence or decreased adherence to treatment, ultimately increasing morbidity and mortality. Despite continuous efforts in fighting stigma in these conditions, there is very limited knowledge on the phenomenon of double stigma, meaning the impact of having two stigmatised conditions at the same time.

**Aims:**

To discuss double stigma in mental health with special reference to epilepsy.

**Method:**

Articles were identified through searches in PubMed up to 31 October 2019 using the search terms ‘epilepsy’, ‘psychiatric disorders’, ‘stigma’ and additional material was identified from the authors’ own files and from chosen bibliographies.

**Results:**

Double stigma is gaining attention for other stigmatised medical conditions, such as HIV, however, the literature on epilepsy is almost non-existent and this is quite astonishing given that one in three people with epilepsy have a lifetime diagnosis of a psychiatric condition. Felt (perceived) stigma and psychiatric disorders, particularly depression, create a vicious circle in epilepsy maintaining both, as depression correlates with stigma and vice versa as well as epilepsy and depression serving as bidirectional risk factors. This phenomenon has no geographical and economic boundaries as similar data have been reported for low-income and high-income countries.

**Conclusions:**

Governments and policymakers as well as health services, patients’ organisations, families and the general public need to be aware of the phenomenon of double stigma in order to develop campaigns and interventions tailored for these patients.

## Background

Epilepsy is a serious neurological disorder affecting approximately 50 million people worldwide.^1,2^ In high-income countries, incidence rates range between 40 and 70/100 000 persons/year, with higher rates among children and elderly people.^3^ Incidence rates are much higher in resource-poor countries, being above 120/100 000/year, but even in high-income countries, poor people seem to have a higher incidence.^4,5^ According to the World Health Organization (WHO), epilepsy accounts for about 0.5% of the global burden of all diseases with total annual costs, in Europe in 2004, of approximately 15.5 billion Euros.^6^

People with epilepsy are among the most vulnerable in our society because epilepsy is a hidden disability and remains today a highly stigmatised condition because of ignorance and superstition caused by centuries of misconceptions towards this disorder.^7^ Social stigma is a key aspect of chronic illnesses and negatively affects quality of life. It is characterised by different components, including enacted stigma (external behaviours and discrimination) and felt (perceived) stigma (shame and expectation of discrimination).^8,9^ Stigma leads not only to discrimination and civil and human rights violations but also to poor access to healthcare and decreased adherence to treatment, ultimately increasing morbidity and mortality. Mental illness shares with epilepsy exactly the same issues because of centuries of misbeliefs, ignorance and superstition about mental health problems with similar consequences in terms of discrimination, violation of human rights and poor access to health resources.

## Double stigma

What happens when two highly stigmatised conditions sensitive to cultural themes occur in the same individual? This phenomenon is known as double stigma and it is now getting attention for psychiatric disorders in other stigmatised medical conditions such as, for example, HIV, tuberculosis or obesity.^10^ However, there is still very limited attention to the problem of double stigma in people with mental health issues and epilepsy. In this narrative review, we aimed at discussing the issue of double stigma in mental health with special reference to epilepsy.

## Method

Articles were identified through searches in PubMed up to 31 October 2019 using the search terms ‘epilepsy’, ‘psychiatric disorders’, ‘stigma’. No language restrictions were applied. This search generated 111 abstracts. Articles were selected based on originality and relevance to the present topic. Additional articles were identified from the authors’ own files and from chosen bibliographies.

## Results

### How frequent are mental health problems in epilepsy?

Mental health problems are not uncommon in epilepsy. Data from cross-sectional epidemiological studies show that one in three people with epilepsy have a lifetime history of a psychiatric disorder and all major psychiatric disorders show prevalence rates in people with epilepsy higher than those reported in the general population.^11^ Furthermore, it is now established that there is a bidirectional relationship between epilepsy and psychiatric disorders.^12^

In adults, a meta-analysis of 14 population-based studies showed an overall prevalence of active depression in epilepsy of 23.1% (95% CI 20.6–28.3%) with an increased overall risk of 2.77 (95% CI 2.09–3.67) compared with the general population.^13^ These estimates, however, vary considerably across studies depending on the ascertainment source (i.e. self-report versus screening tools versus structured clinical interviews), countries, regions and settings. A meta-analysis on anxiety disorders in over 3000 people with epilepsy showed a pooled prevalence of 20.2% (95% CI 15.3–26.0) with generalised anxiety disorder being most common (10.2%; 95% CI 7.7–13.5%).^14^ A meta-analysis of 58 studies of psychosis and related disorders showed a pooled prevalence of 5.6% (95% CI 4.8–6.4%) in unselected samples increasing to 7% (95% CI 4.9–9.1%) in people with temporal lobe epilepsy, with a pooled odds ratio for risk of psychosis compared with the general population of 7.8 (95% CI 2.8–21.8).^15^ Data from epilepsy surgery samples suggest an even higher prevalence.^16^ Still, studies in selected samples of patients with drug-resistant epilepsy have shown prevalence rates for psychiatric disorders up to 60%.^17^

Data from children and adolescents with epilepsy are not different despite an obvious emphasis on developmental disorders. A population-based study in 85 children and adolescents (aged 5–15) with active epilepsy in West Sussex in the UK reported a prevalence of attention-deficit hyperactivity disorder (ADHD) of around 33%, autistic spectrum disorder of 21%, depression of 7% and anxiety of 13%.^18^ A nationwide Norwegian registry study in an unselected paediatric population of over 1 000 000 children reported developmental and psychiatric comorbidities in 43% of children with epilepsy, with overall odds ratios (compared with the general population of children) of 10.7 (95% CI 9.5–12.1) for autism, 5.4 (95% CI 4.8–5.9) for ADHD, 2.3 (95% CI 1.8–3.0) for anxiety disorders and 1.8 (95% CI 1.4–2.5) for depression.^19^ A study of 140 adolescents with epilepsy attending a Hong Kong hospital out-patient neurology clinic reported 22.1% rates for depression and 32.8% for anxiety.^20^

### Stigma and double stigma in mental health

Stigma is a complex phenomenon and research in this area involves specialised disciplines including social sciences and social psychology. The French sociologist Emile Durkheim was probably the first to explore stigma as a social phenomenon but research on stigma in mental health probably started in the 1960s with the American sociologist Erwin Goffman,^21^ followed by the publications of Thomas Scheff^22^ and Bruce Link^23^. However, at that time, the debate focused on how psychiatry was organised, and how this was responsible for the stigma and public attitude towards people with mental health problems. Subsequent research on stigma in mental health progressed identifying different types of stigma and social group processes.^24–26^ The labelling, stereotyping, separation from others and consequent status loss are the key elements of stigma and they are ‘relevant only in a power situation that allows them to unfold’.^27,28^ These studies have highlighted how powerful stereotypes are in our society and their deleterious impact primarily on patients themselves. In fact, negative stereotypes are so ingrained in the collective belief that they become an accepted part of people's belief including patients who internalise how society devalues them. This process is a key element of felt (perceived) stigma and it makes patients feel not empowered to change the situation and, ultimately, maintain the stigma itself.

Negative stereotypes about mental illness are well known. A survey investigating beliefs of random Web users around the world showed that only 7% of people believe that psychiatric disorders could be overcome and between 8% (high-income countries) and 16% (low-income countries) of people believe that individuals with mental health issues are more violent than others.^29^ A cross-sectional survey in people with first-episode psychosis or depression investigated levels of discrimination through face-to-face interview.^30^ People with depression reported greater discrimination in regard to neighbours, dating, education, marriage, religious activities, physical health and acting as a parent than participants with first-episode psychosis. Data from a large prospective population study in Norway showed high chances of divorce in couples where one of the two is diagnosed with a mental illness.^31^ A US study comparing fictitious job applications (*n* = 635) listing mental illness versus physical injury, showed significant discrimination for candidates with a history of mental illness, and this discrimination did not differ for jobs that could be done away from an office setting.^32^

The main consequence of stigma is not simply a negative impact on quality of life for people with mental illness, as stigma has deleterious consequences from a clinical perspective serving as a barrier to health-seeking/diagnosis, treatment and adherence.^33^ A systematic review investigating the relationship between mental health-related stigma and help-seeking clearly showed that stigmatising attitudes towards people with a mental illness is associated with less active help-seeking from patients and their relatives.^34^ In this context, people with an already stigmatised condition often do not reveal their mental state to healthcare professionals for fear of being stigmatised further. Also, healthcare professionals dealing with people with epilepsy are often not skilled in detecting psychological symptoms and, even when they do, they often fail to take the necessary action for further assessment, management and referral.^35,36^ For all these reason, people with double stigma have to fight further in order to receive the best possible care (Appendix).

The European Mental Health Action Plan 2013–2020 has specifically included, among the proposed actions, evidence-based anti-stigma activities in high-risk groups.^37^ In 2018, the WHO published the *United Nations Common Position on Ending HIV, TB and Viral Hepatitis through Intersectoral Collaboration*.^38^ Among the shared principles, it is specifically mentioned that ending these epidemics is not just the absence of the disease but also a state of complete, physical, mental and social well-being, acknowledging the issue of double stigma in these patients. It is quite astonishing that none of these documented listed epilepsy among those medical conditions already stigmatised.

### The double stigma of epilepsy and mental health: a complex paradigm

The impact of stigma is immediately evident on the more intimate life domains of people such as cohabitation and marriage. Patients with epilepsy are less likely to be married or more likely to be divorced as compared with people with other chronic medical conditions.^39–41^ A systematic review of the social outcomes of adults with a history of childhood-onset epilepsy showed that only one in three people were in a romantic relationship, one in three lived independently and one in five had children.^42^ In low-income countries, people with epilepsy are particularly vulnerable to discrimination as in many of these countries there are no anti-discrimination laws or policies in place. A study from Tanzania, showed that only one in three people with epilepsy was in education or in paid employment.^43^ However, stigma easily overcomes anti-discrimination laws and policies and problems are very similar in high-income countries where anti-discrimination laws may not be effective. A survey from Australia showed that 47% of patients with epilepsy were able to document unfair treatment in the workplace.^44^ A systematic review of employability in people with epilepsy showed a mean adjusted employment rate of 58% with no significant differences between continents.^45^ Still, this study showed similar employment rates in people with uncontrolled and those with controlled seizures, suggesting that unemployment is likely to be related to non-clinical factors.^45^

The concept of double stigma in epilepsy and mental health is more complex than the simple additional effects of the two stigmatised conditions. In fact, felt (perceived) stigma is an important determinant *per se* of mental health problems in epilepsy, creating a vicious circle.^46,47^ In epilepsy, perceived stigma is a risk factor for the development of mood and anxiety disorders,^48,49^ which, at the same time, are risk factors for perceived stigma, along with other personality features.^50,51^ This phenomenon is present in low-income as well as high-income countries, independently of background, although specific cultures may have greater degrees of stigma. A study from Ethiopia showed that perceived stigma is associated with depression in people with epilepsy.^52^ In the same way, a study from Italy showed that feeling stigmatised correlates with depressive symptoms rather than seizure frequency.^53^ A systematic review of clinical correlates of stigma in adults with epilepsy showed that stigma is associated with depression and anxiety.^54^

Stigma affects not only patients but also their caregivers and has negative consequences on their mental health.^55,56^ A cross-sectional study from the USA showed that caregivers of people with drug-resistant epilepsy experience high perceived stigma and burden levels.^57^ In low- and middle-income countries, 20% of mothers of children with epilepsy feel stigmatised because of their child's neurological condition.^58^ The caregiver's perception of burden, together with the level of family functioning, are indirectly correlated with depressive symptoms in people with epilepsy via the mediating effect of caregiver depression.^59^ The same phenomena are reported also in high-income countries. A UK study showed that mothers of young children with epilepsy report high levels of parenting stress as compared with mothers of children with non-epilepsy related neurodisabilities.^60^

Studies addressing the phenomenon of double stigma in epilepsy are almost non-existent. A study from Brazil showed that cohabiting relatives of people with epilepsy and depression experience more stigma than those with epilepsy only.^61^ A study from Bosnia Herzegovina, showed that patients with epilepsy and depression present with higher levels of stigma as compared with those with epilepsy only.^62^ However, the phenomenon of double stigma is not analysed in detail.

What can be done to address the problem? Anti-stigma campaigns and educational initiatives about epilepsy can have positive effects on well-being and mental health problems. An educational campaign in rural Bolivia aimed at improving knowledge towards epilepsy showed that the improved knowledge and attitude towards epilepsy was associated with reduced stigma, better quality of life and fewer depressive symptoms.^63^ However, as pointed out by a study from the Philippines, these strategies may not always be successful in the context of double stigma if context-specific approaches are not considered.^64^ It is not possible to develop effective campaigns and policies on double stigma in epilepsy and mental health without a clear analysis of the problem and for this reason, further studies on this phenomenon are urgently needed.

The new definition of epilepsy published in 2014 now defines epilepsy as a disorder of the brain characterised not just by recurrent seizures but also by its psychosocial consequences^65^ and the new classification of syndromes published in 2017 recognises comorbidities as an important part of the diagnostic process.^66^ Furthermore, the new WHO global report on epilepsy also mentions the presence of psychiatric disorders in epilepsy and the importance of addressing them.^1^ These necessary preliminary steps are helpful as they lead to the global acknowledgement of mental health problems in people with epilepsy as well as the associated double stigma but significant work still needs to be done in terms of education (for example society, patients, and healthcare providers) and access to care.

## Discussion

Lack of studies on the phenomenon of double stigma represents a major concern and more research in this area is needed. Both quantitative and qualitative studies as well as systematic reviews and meta-analyses are required to better appreciate the key issues that exist for patients with both epilepsy and mental disorders. In fact, future anti-stigma campaigns will need to take into account the phenomenon of double stigma in order to develop targeted and focused interventions.

Stigma is unacceptable and everyone has the right to be protected from it. Counteracting stigma and discrimination is a duty of governments, health services, medical schools, medical societies, patients’ organisations, families and the general public. Advocacy can take many forms and it represents the best way to fight stigma and to empower people with epilepsy and mental health problems.^67^

## Appendix

**Table:** Consequences of double stigma in people with epilepsy and mental illness

Factor	
Public attitude, knowledge and misbeliefs about epilepsy and mental illness	Patients’ reluctance to disclose mental health problems when affected by a stigmatised condition such as epilepsy
	Felt (perceived) stigma leading to increased rates of mood and anxiety disorders creating a vicious circle
Lack of training of neurologists and psychiatrists in identifying and treating psychiatric disorders in epilepsy	Failure to identify, prevent and treat psychiatric disorders in epilepsy
	Lack of integrated campaigns addressing double stigma
Lack of integrated campaigns addressing double stigma	Failure to identify needs of people with double stigma
	Limited knowledge on the double stigma phenomenon
